# Peering beyond the walls of healthcare institutions: a catalyst for innovation

**DOI:** 10.1186/s12913-017-2342-9

**Published:** 2017-07-11

**Authors:** James K. Elrod, John L. Fortenberry

**Affiliations:** 1 Willis-Knighton Health System, 2600 Greenwood Road, Shreveport, LA 71103 USA; 2 LSU Shreveport, 1 University Place, Shreveport, LA 71115 USA

**Keywords:** Innovation, Innovation adoption, Environmental surveillance, Competitive advantage, Healthcare strategy

## Abstract

**Background:**

Healthcare providers operate in a unique industry characterized by pursuit of perhaps the most noble of missions: the delivery of vital health and medical services to those in need. Distinguishing features abound, differentiating the healthcare industry from others, with such facets having the potential to compel those serving in health and medical establishments to focus exclusively on their selected industry. But directing attention solely within can result in missed opportunities, especially regarding innovation. Many innovations which are well suited for healthcare establishments emerge externally, making at least some exposure beyond the healthcare industry essential for institutions desirous of operating on the innovation frontier.

**Discussion:**

True innovation emerges from broad worldviews, allowing healthcare providers to comprehensively understand the current state of the art. With such an understanding, novel tools, techniques, and approaches, regardless of industry of origin, can be examined for their potential to elevate the status and stature of efforts within health and medical establishments. It is this very open, inquisitive mindset that permitted Willis-Knighton Health System to identify and incorporate a range of innovations which originated outside of the healthcare industry. Its embracement of and associated successes with the repurposing approach known as adaptive reuse, the delivery of complex medical services via centers of excellence, and the structuring of operations using the hub-and-spoke organization design, for example, would never have occurred had executives not directed attention externally in search of innovations that could be used within.

**Conclusions:**

Innovations offer key pathways for healthcare providers to enhance the depth and breadth of health and medical services offered in their establishments and communities. By peering beyond the walls of healthcare institutions, providers amplify opportunities to discover novel methods and approaches that potentially can be transferred into their own organizations, benefiting themselves and their patient populations.

## Background

The healthcare industry is unlike any other industry in existence. Characterized by what many would consider to be the most noble of missions—the delivery of health and medical services to those in need—physicians, nurses, administrators, and others endeavor each and every day in their various establishments to provide the very best of care and attention for their patients. Accomplishing this important mission is trying, but it is made more difficult by the nature of the healthcare environment which features notable challenges, including perpetual change, keen competition, ever-evolving reimbursement systems, and extensive regulatory oversight [1, 2]. The end result of all of this is a most unique industry [3].

The special nature of the healthcare industry makes for very intriguing and interesting work environments, but it also can foster very compartmentalized, insular mindsets [4, 5]. Such mindsets set the stage for those serving in health and medical establishments to concentrate on developments within their given industry of operation, often so much so that the greater environment of business and industry is neglected [6, 7]. In fact, opportunities abound for this to occur. Healthcare personnel typically work hand-in-hand with others engaged in like pursuits, hold memberships in healthcare-related professional societies, subscribe to newsletters and other publications which focus on health and medical care, and attend conferences centered on health matters. These and related activities nurture a high degree of industry solidarity. They also aid in perpetuating inward-directed thought, something that at least somewhat characterizes the healthcare industry [4, 5, 8].

This concentrated, devoted focus on healthcare industry matters, in and of itself, is not inappropriate. Those engaged in the delivery of healthcare services must be operating in top form if they wish to benefit their given patient populations. This, of course, requires extensive exposure to healthcare-specific knowledge sources, something the industry is especially adept at providing through employers, consultants, professional societies, publications, and the like. However, on some fronts, particularly that of innovation, insular mindsets which focus solely on the healthcare industry can be problematic as they diminish opportunities for exposure to novel advancements, insights, and know-how emerging in other industries [4, 9]. While some might disregard such innovations, considering them to be nontransferable due to the unique nature of the healthcare industry, this isn’t always the case. Some externally-developed innovations almost certainly will be well suited for health and medical establishments, but unless one is open to looking beyond the walls of healthcare institutions, these opportunities will be missed [4, 8, 9].

## Discussion

True innovation emerges from broad worldviews, as such expansive perspectives afford a comprehensive understanding of the current state of the art. This requires abandoning insular mindsets in favor of a more balanced approach which directs significant attention toward the healthcare industry and at least periodic attention toward the greater environment of business and industry. This approach quite obviously continues to prioritize health and medical matters, but it importantly opens a window to the outside world, permitting opportunities for exposure to potentially transferable innovations which could elevate and enhance healthcare operations and ultimately benefit patient populations [4, 9, 10].

This open window to the outside world has made a profound impact on the state of innovation at Willis-Knighton Health System. Historically, whenever executives desired service enhancements, sought increased productivity, or encountered pressing dilemmas, innovative solutions were sought from both within and outside of the healthcare industry. This broad, inquisitive mindset increased pools of options for any given decision point, permitting optimal approaches to be selected and advanced for the benefit of the institution and its patients. It also afforded significant competitive advantages, especially in cases where the given innovations emerged from industries beyond the domain of healthcare, as many rivals didn’t possess the open window perspectives required for exposure to these innovations [10].

Willis-Knighton Health System’s embracement of and associated successes with the repurposing approach known as adaptive reuse (ca. 1970s-present), the delivery of complex medical services via centers of excellence (ca. 1980s-present), and the structuring of operations using the hub-and-spoke organization design (ca. 1980s-present), for example, would never have occurred had executives not directed attention externally in search of innovations that could be used within. These particular innovations each originated outside of the healthcare industry and while they now have either acquired, or are in the process of acquiring, a firm place within the industry, that wasn’t the case when Willis-Knighton Health System turned to the given practices.

In respective eras of adoption, Willis-Knighton Health System’s executives had few insights regarding associated applications within the healthcare industry, but they had confidence that the innovations would transfer successfully and deliver results similar to those experienced by institutions in other industries which were making use of the given practices. The system’s pioneering steps introducing these innovations into its operations carried risk, but also the potential for rewards. All told, the adopted innovations yielded benefits far exceeding expectations, with the resulting approaches continuing to deliver value to this day, supplying excellent examples of the successful transfer of innovations emerging outside of the healthcare industry into operating medical establishments.

Ultimately, peering beyond the walls of healthcare institutions serves as a catalyst for innovation, increasing opportunities for exposure to potentially transferrable insights and approaches that healthcare institutions might be able to make their own [4, 11]. Operationalizing this complementary layer of environmental surveillance can take many forms, some being formal and others less so.

Willis-Knighton Health System’s particular approach rests with its executive staff and associated directives requiring each member to stay abreast of environmental developments, whether originating within or outside of the healthcare industry. Beyond maintaining keen environmental awareness, staff members must also anticipate the potential impact of developments on the institution. Useful tools for such endeavors include the PEST analysis, which involves the identification and assessment of political, economic, social, and technological environmental developments, and the SWOT analysis, which entails the investigation and identification of institutional strengths, weaknesses, opportunities, and threats [12].

This charge ultimately forces an outward focus that extends well beyond the walls of Willis-Knighton Health System and even the greater healthcare industry. Developments are shared regularly in executive staff meetings and, when necessary, more deeply investigated with the assistance of consultants and members of the academic research community. Importantly, executives understand that productive tools, techniques, approaches, and pathways can emerge from virtually anywhere, with this philosophy encouraging broad worldviews that set the stage for success across many areas, including the pursuit and realization of innovation. Open windows to the outside world, as discussed earlier, are mandatory at Willis-Knighton Health System.

It should be noted that creativity is essential for successfully pursuing and evaluating innovations emerging outside of the healthcare industry [10, 11]. Some innovations encountered indeed may offer no benefit to healthcare establishments, but others will demonstrate potential. Of those with potential, some can be transferred into healthcare operations directly without the need for any modifications. Willis-Knighton Health System’s adoption of adaptive reuse, for example, represented a direct transfer into the healthcare industry from its origins in retail, hospitality, and housing sectors. No modifications whatsoever were required for immediate applicability in healthcare settings.

On other occasions, innovations encountered will not be directly transferrable, but can be altered to make them suitable for use within healthcare institutions. Willis-Knighton Health System’s adoption of the center of excellence delivery model and incorporation of the hub-and-spoke organization design illustrate this nicely. Centers of excellence as applied in research and technology institutions supplied helpful conceptual frameworks, but modifications were necessary for this particular model to be suitable for use in healthcare delivery environments. Similarly, the hub-and-spoke organization design, originating in the transportation industry, required adaptations to arrive at a framework which fit healthcare operations.

Indeed, some innovations which appear to be unsuitable for use in healthcare establishments may very well be adapted successfully with ingenuity and associated modifications, placing a premium on creative thought whenever evaluating the transfer potential of externally-derived innovations. Coupling such creativity with environmental surveillance systems that aren’t confined to healthcare industry examinations can yield groundbreaking innovations, such as those achieved by Willis-Knighton Health System.

## Conclusions

Innovations offer key pathways for healthcare providers to enhance the depth and breadth of health and medical services offered in their associated establishments and communities. Those healthcare institutions desiring to be part of the innovation frontier must be especially vigilant in their quests to identify novel tools, techniques, and approaches which possess the potential to elevate the status and stature of efforts within their establishments. In such pursuits, insular mindsets which focus solely on the healthcare industry are detrimental, as they limit exposure to the full range of advancements occurring in the greater environment of business and industry. Healthcare industry surveillance simply isn’t enough; attention also must be directed externally.

By peering beyond the walls of healthcare institutions, providers amplify opportunities to discover novel methods and approaches that potentially can be transferred into their own organizations, benefiting themselves and their patient populations. This catalyst for innovation, derived from a complementary layer of environmental surveillance which features a window to the outside world, has afforded Willis-Knighton Health System with significant competitive advantages, operational efficiencies and effectiveness, and much more, illustrating the many benefits associated with embracing a broad and balanced approach in quests to identify and realize innovations.
